# Supplementary figures and images for: Oral toxicity of arjunolic acid on hematological, biochemical and histopathological investigations in female Sprague Dawley rats
Source: PeerJ. 2019 Nov 22;7:e8045. doi: 10.7717/peerj.8045 (PMC6876537; doi:10.7717/peerj.8045)

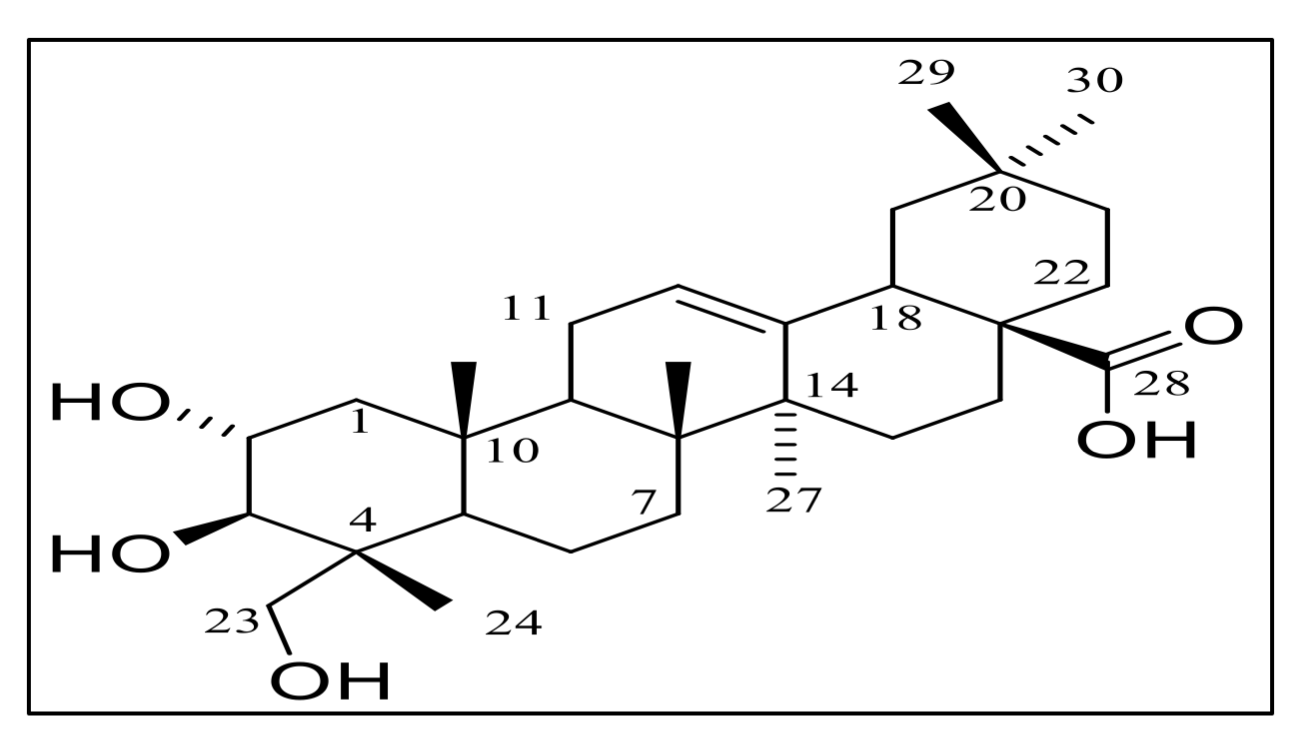

Supplement: Supplemental Information 1 [file peerj-07-8045-s001.png]
